# Age, breed, sex and diet influence serum metabolite profiles of 2000 pet dogs

**DOI:** 10.1098/rsos.211642

**Published:** 2022-02-16

**Authors:** Jenni Puurunen, Claudia Ottka, Milla Salonen, Julia E. Niskanen, Hannes Lohi

**Affiliations:** ^1^ PetBiomics Ltd, 00300 Helsinki, Finland; ^2^ Department of Veterinary Biosciences, University of Helsinki, 00014 Helsinki, Finland; ^3^ Department of Medical and Clinical Genetics, University of Helsinki, 00014 Helsinki, Finland; ^4^ Folkhälsan Research Center, 00290 Helsinki, Finland

**Keywords:** canine metabolism, canine physiology, nuclear magnetic resonance metabolomics, metabolites

## Abstract

As an individual's metabolism reflects health and disease states well, metabolomics holds a vast potential in biomedical applications. However, normal physiological factors, such as age, can also influence metabolism, challenging the establishment of disease-specific metabolic aberrations. Here, we examined how physiological and diet-related factors drive variance in the metabolism of healthy pet dogs. We analysed 2068 serum samples using a canine nuclear magnetic resonance (NMR) spectroscopy-based metabolomics platform. With generalized linear models, we discovered that age, breed, sex, sterilization, diet type and fasting time significantly affected the canine metabolite profiles. Especially, breed and age caused considerable variation in the metabolite concentrations, and breeds with very different body conformations systematically differed in several lipid measurands. Our results enhance the understanding how normal physiological factors influence canine metabolism, aid accurate interpretation of the NMR results, and suggest the NMR platform might be applied in identifying aberrations in nutrient absorption and metabolism.

## 1. Background

Metabolomics offers an extensive snapshot of an individual's current state of metabolism by measuring the intermediate and end product molecules of metabolism, metabolites [1,2]. The metabolome is affected by several internal factors, including gene and protein activity, and external factors, including diet and environmental effects, and their interplay together with host–microbiota interactions. This places metabolomics in a key position for understanding multi-factorial diseases and the complex effects, how physiological factors can affect health [3]. However, it is important to distinguish metabolic changes caused by physiological variation from pathological disease-related metabolic alterations [4,5].

There is a lack of large-scale metabolomics studies investigating the global effects of physiological factors, such as age, breed and sex, on canine metabolism. Due to artificial selection, there are more than 400 genetically, morphologically, physiologically and behaviourally different dog breeds that represent closed genetic populations with high intra-breed homogeneity and high interbreed heterogeneity [6–9], suggesting that breeds would differ also metabolically [10–12]. Previous studies have identified variation in haematological and clinical chemistry analytes due to breed [13–20] and age [14,21–26], for example. In humans, sex differences in metabolism have been demonstrated [5,27,28], suggesting that similar effects could be evident also in dogs. Furthermore, nutritional factors, such as consumed diet [29] and time from the last meal [30,31], may have profound effects on metabolite measurand levels, especially lipid concentrations.

Recently, we have developed and validated a nuclear magnetic resonance (NMR) spectroscopy-based canine metabolomics platform quantitating 123 measurands in serum and plasma [32]. The high-throughput and reproducibility of the method, together with the quantitative nature, make the platform highly promising for clinical and research usage in veterinary medicine. Here, we aimed to identify how age, breed, sex, sterilization, body size, diet and fasting time before blood sampling drive variance in the metabolism of healthy Finnish pet dogs using the NMR metabolomics approach. Understanding of how normal physiological factors influence the metabolism of healthy individuals is crucial for several reasons: it is a prerequisite for identifying disease-associated metabolic changes and vital for appropriate metabolomics study designs. In addition, it can facilitate translational research using the dog as a model animal. Moreover, it is required for accurate interpretation of laboratory results in veterinary diagnostics.

## 2. Methods

### 2.1. Sample collection

The samples were derived from an earlier study cohort [32]. Briefly, blood samples were collected across Finland from 4816 client-owned dogs in 2017–2018. The blood samples were drawn by cephalic venipuncture, allowed to clot for a minimum of 30 min, and centrifuged at 3000*g* for 10 min to separate the serum. The serum samples were immediately refrigerated and stored at −80°C until NMR analysis. The dog owners completed questionnaires regarding the current health, diet, exercise, stress and reproductive state of the dog. All dog owners participated in the study voluntarily and gave informed consent.

### 2.2. NMR metabolomics analysis

We used a validated and fully automated canine-specific NMR metabolomics technology, which quantitates 123 measurands (electronic supplementary material, table S1) from serum samples [32]. Details of the NMR method are provided elsewhere [32,33].

### 2.3. Data preprocessing

Data preprocessing was conducted in Microsoft Excel and the following statistical analyses in R v. 4.0.2 [34].

#### 2.3.1. Study cohort

To ensure a balanced and high-quality study population, only a certain subset of the aforementioned study cohort [32] was used in this study. Firstly, we only included serum samples separated from whole blood within 45 min from sampling to avoid the effects of prolonged red blood cell contact. In addition, we included only dogs reported to be healthy according to the owner: dogs reported to suffer from any systemic disease or being treated for a certain condition during the time of blood sampling were excluded. For example, dogs were not excluded solely based on results from routine X-rays of the joints and back, but they were excluded if the dogs were reported to show symptoms or were treated. This approach was chosen since the definition of a disease always requires the presence of symptoms. Dogs diagnosed with minor eye diseases, such as distichiasis, that were untreated were not excluded since their systemic metabolic effects were considered negligible. Additionally, we only included dogs that were not in any of the following hormonally active states during the time of sampling: pro-oestrus and oestrus, pseudopregnancy, pregnancy or lactation. Four dogs had missing values in most measurands and thus, were excluded. This resulted in a sample size of 3325 dogs (electronic supplementary material, figure S1). Moreover, to assure optimal study groups for statistical analyses, we excluded dogs with missing information in age, sex, sterilization status, breed, body size, diet and fasting time. Additionally, some categories of the diet variable had very low sample sizes (homemade food, leftover food and something else), and thus, dogs consuming these diets were excluded. As a result, the diet variable contained four categories: raw food, dry food, raw + dry food and several food types, and the analytical sample size was 3030 dogs. Finally, we wanted to ensure adequate breed-specific sample sizes and thus, only breeds with more than 35 individuals were included. This resulted in an analytical sample size of 2069 dogs in 22 breeds (American Staffordshire Terrier (AST), Australian Kelpie (AUK), Australian Shepherd (AUS), Belgian Shepherd Dog Groenendael (BSG), Bernese Mountain Dog (BER), Border Collie (BC), Chihuahua (both coat types; CHI), Cirneco d'ell Etna (CDE), French Bulldog (FBD), German Shepherd Dog (GSD), Golden Retriever (GR), Great Dane (GD), Greyhound (GH), Jack Russell Terrier (JRT), Labrador Retriever (LR), Leonberger (LEO), Norrbottenspitz (NBS), Parson Russell Terrier (PRT), Schapendoes (SCP), Spanish Waterdog (SWD), Staffordshire Bullterrier (SBT) and Whippet (WH)) (electronic supplementary material, figure S1 and table S2). Of this study population, 571 dogs (25.6%) were previously used in the determination of serum reference intervals in a study of Ottka *et al*. [32].

#### 2.3.2. Missing value imputation

Many measurands had several missing values. Therefore, metabolites with more than 50% of their values missing (XL-VLDL-CE, XL-VLDL-FC, XL-VLDL-PL) were excluded from further analysis (electronic supplementary material, figure S1). For metabolites with less than 50% of their values missing, random forest imputation was used to replace missing values. Package ‘missForest’ (v. 1.4) [35] was used for imputation with the default values of missForest function (maximum number of iterations was set to 10 and number of trees to 100). The normalized root mean squared error (NRMSE) value, describing the true imputation error of the iteration was 0.00061, indicating good performance of the RF imputation on the data.

### 2.4. Statistical analyses

The distributions of continuous variables were assessed by histograms, and due to skewed distributions, generalized linear models (GLM) were used. The distributions and link functions were determined for each model by inspecting the residual plots of the models with packages ‘boot’ [36] and ‘rcompanion’ (v. 2.3.25) [37]. For most measurands, gamma distribution with a log link function was used (electronic supplementary material, data S1). VLDL triglycerides was excluded from the GLM analyses as we could not find a distribution and a link function that would have fitted the data adequately (electronic supplementary material, figure S1). Therefore, the final number of the studied measurands was 119.

We built the models for each measurand separately, with the measurand level serving as the continuous dependent variable. Age, sex, breed, sterilization, body size, diet and fasting time were selected as possible explanatory variables for the models (electronic supplementary material, table S3). We used a forward stepwise Akaike information criterion (AIC) model selection approach by adding variables one by one to obtain the final models with the best fit, starting with base models including age and sex as explanatory variables. The body size variable had high multi-collinearity with breed and was thus excluded from the analyses. The model selection was performed recursively with an in-house model selection loop written in R [38]. The final models and AIC selection processes for each metabolite measurand are shown in electronic supplementary material, data S1.

We assessed model fit carefully. To test the linearity assumption of continuous explanatory variables (i.e. age), we fitted generalized additive models with the package ‘gam’ (v. 1.20) [39] after model selection. If the assumption was not met, we added quadratic age (age^2^) in the final model. To inspect possible outliers and influential data points in the models, we plotted standardized residuals with packages ‘broom’ (v. 0.7.0) [40], ‘dplyr’ (v. 1.0.1) [41], ‘ggplot2’ [42], ‘car’ [43], ‘gplots’ (v. 3.0.4) [44], ‘boot’ [36] and ‘rcompanion’ (v. 2.3.25) [37]. One dog (4 years old intact female Jack Russell Terrier) was excluded as an outlier from all the models due to highly deviant values in several measurands. Thus, the final sample size used in the GLM models was 2068 dogs in 22 breeds (electronic supplementary material, figure S1). Finally, we tested multi-collinearity of the variables and autocorrelation of the residuals with packages ‘car’ [43] and ‘lmtest’ [45], respectively. After fitting the models, the overall effects of all explanatory variables were derived from an analysis of variance (ANOVA) with the package ‘car’ [43]. With package ‘emmeans’ (v. 1.4.8) [46], we calculated the estimated marginal means for categorical explanatory variables (adjusting for other variables in the models). Additionally, the effects of continuous explanatory variables (adjusting for other variables in the models) were obtained with the package ‘effects’ [43,47]. All *p*-values were corrected for Benjamini–Hochberg false discovery rate (FDR) due to the high number of comparisons [48], and the significance cut-off was set at *p*-value less than 0.05. We prepared figures to illustrate the effects of explanatory variables on the metabolite measurands with ‘ggplot2’ [42], ‘ggthemes’ (v. 4.2.0) [49], ‘cowplot’ (v.1.0.0) [50], ‘gridExtra’ (v. 2.3) [51], and ‘ggpubr’ (v. 0.4.0) [52]. Circular heatmaps, created to summarize and simplify the results, were generated with packages ‘ComplexHeatmap’ [53] and ‘circlize’ [54]. The figures were finalized in Inkscape Project [55].

## 3. Results

### 3.1. Study cohort and demographics

Our study cohort comprised 2068 dogs reported healthy by the dog owners. The cohort included 22 dog breeds, BC having the largest sample size (*N* = 170) and NBS the smallest sample size (*N* = 36) (electronic supplementary material, table S2). The age of the dogs varied from 1 month to 16 years, with a mean of 3.10 years (s.d. = 3.10). Females comprised 54.8% of the population, and 84.9% of the dogs were intact at the time of blood sampling.

### 3.2. Generalized linear models

**Figure 1.**
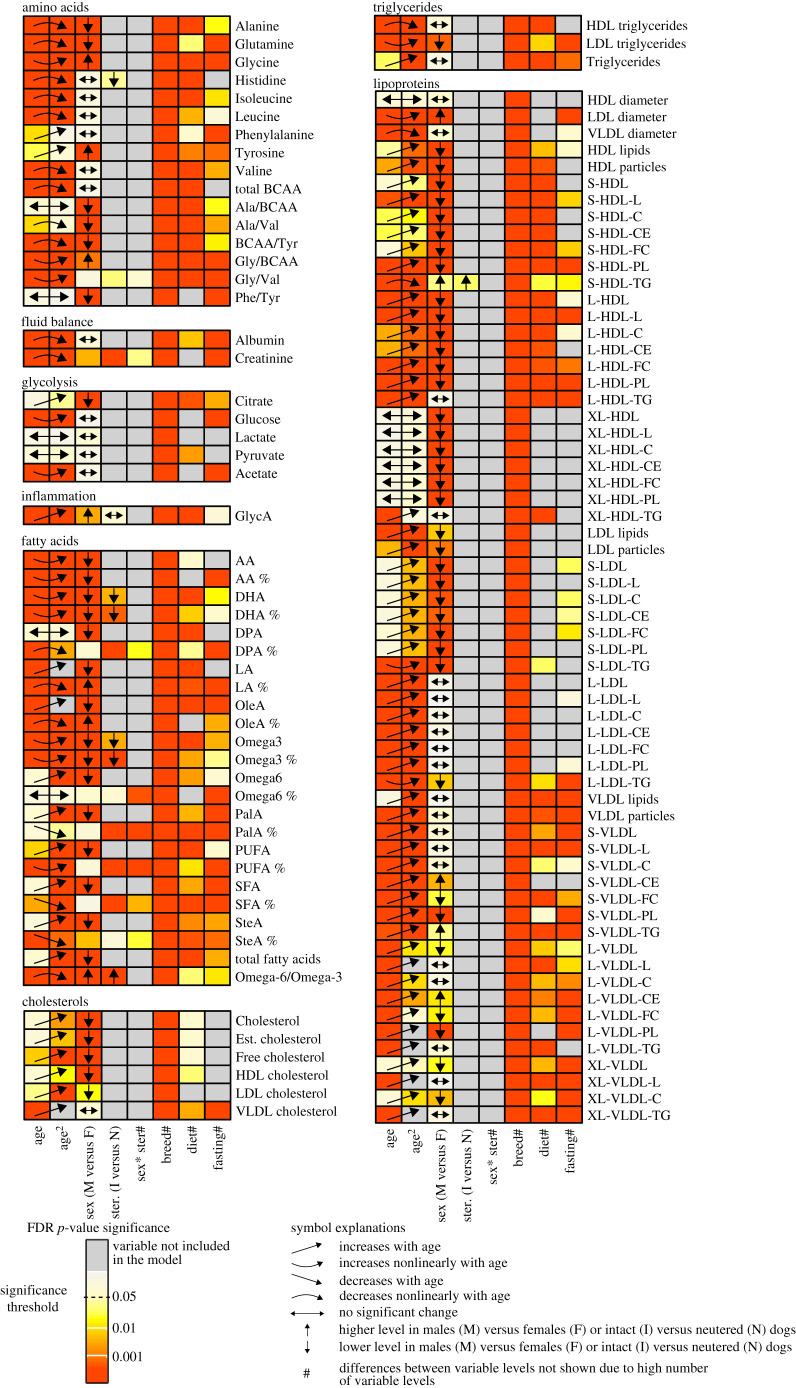
A summary of the overall associations between measurands and physiological and diet-related variables coloured by FDR *p*-values. Additionally, the age, sex and sterilization differences are summarized in the figure. The sex×sterilization interaction, breed, diet and fasting time differences, however, are not summarized due to the high number of variable levels and thus, complexity of the differences. The colour scale indicates the significance of the association between the measurand and variable: light yellow *p* > 0.05, yellow *p* = 0.01–0.05, orange *p* = 0.001–0.01 and red *p* < 0.001. Grey colour indicates that the variable did not affect metabolite measurand level and was thus not included in the multi-variable generalized linear model of that particular measurand. M, male; F, female; ster, sterilization; I, intact; N, neutered; sex*ster, interaction between sex and sterilization. The full names of the measurands are reported in electronic supplementary material, table S1.

**Figure 2.**
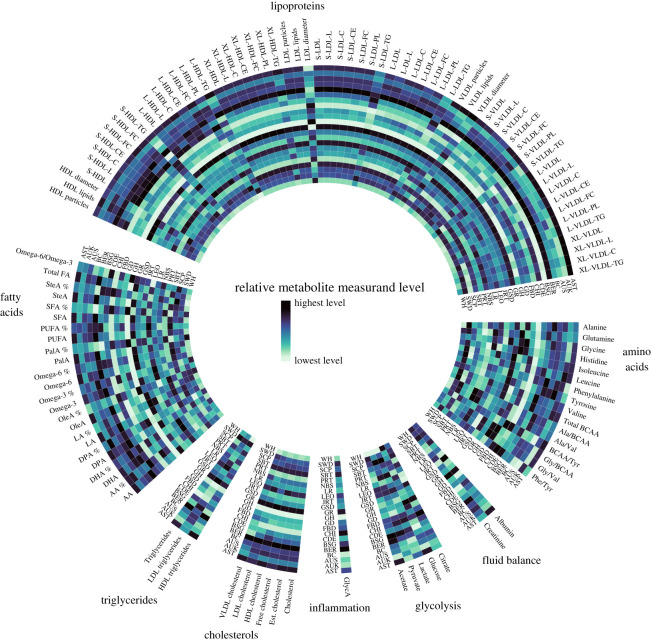
A graphical illustration of the relative differences in the measurand levels between different dog breeds. The circular tracks from outside to inside: breeds (in alphabetical order) by different measurands. In each different measurand, the darkest blue colour illustrates the breed with the highest level, and the lightest green colour illustrates the breed with the lowest level of that particular measurand. AST, American Staffordshire Terrier; AUK, Australian Kelpie; AUS, Australian Shepherd; BSG, Belgian Shepherd Dog Groenendael; BER, Bernese Mountain Dog; BC, Border Collie; CHI, Chihuahua (both coat types); CDE, Cirneco d'ell Etna; FBD, French Bulldog; GSD, German Shepherd Dog; GR, Golden Retriever; GD, Great Dane; GH, Greyhound; JRT, Jack Russell Terrier; LR, Labrador Retriever; LEO, Leonberger; NBS, Norrbottenspitz; PRT, Parson Russell Terrier; SCP, Schapendoes; SWD, Spanish Waterdog; SBT, Staffordshire Bullterrier; WH, Whippet. The full names of the measurands are reported in electronic supplementary material, table S1.

We investigated the effects of breed, age, sex, sterilization, diet and fasting time before blood sampling on a total of 119 measurands: nine primary, one combined and six ratio amino acid measurands; two fluid balance-related measurands; five glycolysis-related measurands; one inflammation-related measurand; six cholesterol measurands; three triglyceride measurands; seven fatty acids in their absolute and relative (indicated by % sign) units, four combined fatty acids measurands in their absolute and relative (%) units, the total concentration of fatty acids, and one ratio fatty acid measurand; and 24 high-density lipoprotein (HDL), 17 low-density lipoprotein (LDL) and 21 very-low-density lipoprotein (VLDL) particle measurands (the measurands are listed in electronic supplementary material, table S1 together with their abbreviations and units). The overall significances and the main effects of age, sex, sterilization, breed, diet and fasting time on measurand levels are summarized in figure 1, and more detailed breed differences in figure 2. Electronic supplementary material, figures S2–S41 present the precise effects of all studied variables on all measurands.

#### 3.2.1. Amino acids

Breed had a significant effect on all amino acid measurands, and CHI had relatively low levels of several measurands (electronic supplementary material, figure S2, data S2). The largest pairwise breed difference was observed between AUS and CHI in tyrosine concentration (*Z* = 15.23, d.f. = 1, *p* = 3.815 × 10^−50^) (electronic supplementary material, table S4). Age significantly affected all other amino acid measurands but Ala/BCAA and Phe/Tyr, and most associations were nonlinear (table 1; electronic supplementary material, figure S10). Females had higher alanine, glutamine, Ala/BCAA, Ala/Val, BCAA/Tyr and Phe/Tyr, whereas males had higher glycine, tyrosine and Gly/BCAA (electronic supplementary material, figure S18, data S2). Sex and sterilization had significant interaction on Gly/Val as intact males had higher levels than intact females (*Z* = 4.14, d.f. = 1, *p* = 0.0001) and neutered males (*Z* = 2.45, d.f. = 1, *p* = 0.026). Diet affected all other amino acid measurands than Phe/Tyr. The individual branched-chain amino acids isoleucine, leucine and valine, as well as the total BCAA concentration, were higher in the raw food group versus other diet types, whereas in other amino acid measurands, the highest measurand concentrations were usually in the dry food group (electronic supplementary material, figure S26, data S2). Fasting time influenced all other amino acid measurands than histidine and total BCAA (electronic supplementary material, figure S34, data S2).

**Table 1.** Variables best explaining variation in amino acid measurand levels in dogs. *p*-values were controlled for false discovery rate (FDR). Significant effects are indicated in bold (FDR *p* < 0.05). *N* = 2068. ster, sterilization; sex*ster, interaction of sex and sterilization. The full names of the measurands are reported in electronic supplementary material, table S1.

	age	age^2^	sex	breed	diet	fasting	ster	sex*ster
variable	d.f. = 1	d.f. = 1	d.f. = 1	d.f. = 21	d.f. = 3	d.f. = 3	d.f. = 1	d.f. = 1
Ala	*F =* 151.50	*F =* 65.20	*F* = 56.91	*F* = 14.96	*F* = 17.37	*F* = 4.27		
	***p*** = **2.64 × 10^−15^**	***p*** = **1.29 × 10^−14^**	***p*** = **6.52 × 10^−13^**	***p*** = **2.64 × 10^−15^**	***p*** = **2.74 × 10^−10^**	***p*** = **0.0105**		
Gln	*F =* 32.32	*F* = 25.91	*F* = 24.86	*F* = 29.19	*F* = 3.52	*F* = 26.19		
	***p*** = **7.73 × 10^−08^**	***p*** = **1.64 × 10^−06^**	***p*** = **2.71 × 10^−06^**	***p*** = **2.64 × 10^−15^**	***p*** = **0.0266**	***p*** = **2.64 × 10^−15^**		
Gly	*F* = 230.13	*F* = 147.72	*F* = 16.62	*F* = 16.95	*F* = 13.58	*F* = 46.02		
	***p*** = **2.64 × 10^−15^**	***p*** = **2.64 × 10^−15^**	***p*** = **0.0001**	***p*** = **2.64 × 10^−15^**	***p*** = **4.74 × 10^−08^**	***p*** = **2.64 × 10^−15^**		
His	*F* = 57.43	*F* = 38.61	*F* = 0.75	*F* = 8.89	*F* = 12.05		*F* = 5.41	
	***p*** = **5.10 × 10^−13^**	***p*** = **3.84 × 10^−09^**	*p* = 0.4666	***p*** = **2.64 × 10^−15^**	***p*** = **3.77 × 10^−07^**		***p*** = **0.0356**	
Ile	*F* = 80.34	*F* = 41.07	*F* = 0.69	*F* = 7.81	*F* = 7.66	*F* = 4.55		
	***p*** = **2.64 × 10^−15^**	***p*** = **1.19 × 10^−09^**	*p* = 0.4847	***p*** = **2.64 × 10^−15^**	***p*** = **0.0001**	***p*** = **0.0073**		
Leu	*F* = 238.14	*F* = 136.95	*F* = 1.05	*F* = 4.56	*F* = 5.42	*F* = 1.55		
	***p*** = **2.64 × 10^−15^**	***p*** = **2.64 × 10^−15^**	*p* = 0.3816	***p*** = **2.23 × 10^−10^**	***p*** = **0.0024**	*p* = 0.2668		
Phe	*F* = 8.75	*F* = 0.35	*F* = 0.43	*F* = 8.66	*F* = 2.73	*F* = 24.63		
	***p*** = **0.0067**	*p* = 0.6219	*p* = 0.5885	***p*** = **2.64 × 10^−15^**	*p* = 0.0696	***p*** = **1.33 × 10^−14^**		
Tyr	*F* = 6.73	*F* = 2.13	*F* = 48.92	*F* = 17.45	*F* = 6.44	*F* = 6.78		
	***p*** = **0.0183**	*p* = 0.2012	***p*** = **2.84 × 10^−11^**	***p*** = **2.64 × 10^−15^**	***p*** = **0.0006**	***p*** = **0.0004**		
Val	*F* = 107.20	*F* = 46.26	*F* = 0.78	*F* = 8.85	*F* = 18.76	*F* = 5.63		
	***p*** = **2.64 × 10^−15^**	***p*** = **1.00 × 10^−10^**	*p* = 0.4550	***p*** = **2.64 × 10^−15^**	***p*** = **4.07 × 10^−11^**	***p*** = **0.0019**		
total BCAA	*F* = 156.14	*F* = 75.99	*F* = 0.33	*F* = 6.92	*F* = 12.51			
	***p*** = **2.64 × 10^−15^**	***p*** = **2.64 × 10^−15^**	*p* = 0.6366	***p*** = **2.64 × 10^−15^**	***p*** = **2.04 × 10^−07^**			
Ala/BCAA	*F* = 3.82	*F* = 0.70	*F* = 45.66	*F* = 8.41	*F* = 39.72	*F* = 4.23		
	*p* = 0.0811	*p* = 0.4833	***p*** = **1.34 × 10^−10^**	***p*** = **2.64 × 10^−15^**	***p*** = **2.64 × 10^−15^**	***p*** = **0.0110**		
Ala/Val	*F* = 8.93	*F* = 4.05	*F* = 39.56	*F* = 7.42	*F* = 43.83	*F* = 6.14		
	***p*** = **0.0061**	*p* = 0.0721	***p*** = **2.44 × 10^−09^**	***p*** = **2.64 × 10^−15^**	***p*** = **2.64 × 10^−15^**	***p*** = **0.0010**		
BCAA/Tyr	*F* = 83.23	*F* = 47.53	*F* = 44.08	*F* = 9.38	*F* = 24.86	*F* = 4.42		
	***p*** = **2.64 × 10^−15^**	***p*** = **5.51 × 10^−11^**	***p*** = **2.83 × 10^−10^**	***p*** = **2.64 × 10^−15^**	***p*** = **9.71 × 10^−15^**	***p*** = **0.0087**		
Gly/BCAA	*F* = 425.10	*F* = 246.47	*F* = 14.01	*F* = 14.14	*F* = 22.97	*F* = 29.61		
	***p*** = **2.64 × 10^−15^**	***p*** = **2.64 × 10^−15^**	***p*** = **0.0005**	***p*** = **2.64 × 10^−15^**	***p*** = **1.30 × 10^−13^**	***p*** = **2.64 × 10^−15^**		
Gly/Val	*F* = 375.96	*F* = 219.33	*F* = 0.10	*F* = 14.41	*F* = 26.15	*F* = 24.68	*F* = 5.85	*F* = 3.31
	***p*** = **2.64 × 10^−15^**	***p*** = **2.64 × 10^−15^**	*p* = 0.7967	***p*** = **2.64 × 10^−15^**	***p*** = **2.64 × 10^−15^**	***p*** = **1.24 × 10^−14^**	***p*** = **0.0285**	*p* = 0.1063
Phe/Tyr	*F* = 0.87	*F* = 0.13	*F* = 38.23	*F* = 6.20		*F* = 32.68		
	*p* = 0.4285	*p* = 0.7691	***p*** = **4.60 × 10^−09^**	***p*** = **2.64 × 10^−15^**		***p*** = **2.64 × 10^−15^**		

#### 3.2.2. Fluid balance

GH had high albumin and creatinine levels, and the creatinine concentration of GH even exceeded the upper reference limit (RL) (electronic supplementary material, figure S3). The largest pairwise breed differences were between GD and JRT in albumin (*Z* = −11.59, d.f. = 1, *p* = 2.171 × 10^−29^), and between CHI and GH in creatinine (*Z* = −24.94, d.f. = 1, *p* = 7.432 × 10^−133^) (electronic supplementary material, table S4, data S2). In both measurands, dogs from 4 to 8 years had the highest concentrations, after which the concentrations dropped even below the lower RL after 14 years of age (table 2; electronic supplementary material, figure S11). There was no sex difference in albumin, but the interaction of sex and sterilization affected creatinine as neutered males had a higher concentration when compared with neutered females (*Z* = 3.30, d.f. = 1, *p* = 0.002) and intact males (*Z* = 4.44, d.f. = 1, *p* = 3.046 × 10^−5^) (electronic supplementary material, figure S19, data S2). Dogs eating several food types had lower albumin levels than dogs eating raw food or raw + dry food (electronic supplementary material, figure S27, data S2). Both measurands were affected by the fasting time (electronic supplementary material, figure S35, data S2).

**Table 2.** Variables best explaining variation in concentrations of fluid balance-related and glycolysis-related measurands, and glycoprotein acetyls (GlycA) in dogs. *p*-values were controlled for false discovery rate (FDR). Significant effects are indicated in bold (FDR *p* < 0.05). *N* = 2068. ster, sterilization; sex*ster, interaction of sex and sterilization.

variable	age	age^2^	sex	breed	diet	fasting	ster	sex*ster
	d.f.=1	d.f. = *f* = 1	d.f. = 1	d.f. = = 21	d.f. = 3	d.f. = 3	d.f. = 1	d.f. = 1
**fluid-balance**								
albumin	*F* = 364.14	*F* = 285.66	*F* = 1.45	*F* = 12.95	*F* = 4.99	*F* = 25.19		
	***p*** = **2.64 × 10^−15^**	***p*** = **2.64 × 10^−15^**	*p* = 0.2993	***p*** = **2.64 × 10^−15^**	***p*** = **0.0042**	***p*** = **6.16 × 10^−15^**		
creatinine	*F* = 344.02	*F* = 262.75	*F* = 10.87	*F* = 53.29		*F* = 60.70	*F* = 19.73	*F* = 5.61
	***p*** = **2.64 × 10^−15^**	***p*** = **2.64 × 10^−15^**	***p*** = **0.0023**	***p*** = **2.64 × 10^−15^**		***p*** = **2.64 × 10^−15^**	***p*** = **3.20 × 10^−5^**	***p*** = **0.0322**
**glycolysis**								
citrate	*F* = 0.73	*F* = 5.42	*F* = 28.60	*F* = 16.70	*F* = 7.22	*F* = 5.67		
	*p* = 0.4735	***p*** = **0.0354**	***p*** = **4.55 × 10^−7^**	***p*** = **2.64 × 10^−15^**	***p*** = **0.0002**	***p*** = **0.0018**		
glucose	*F* = 421.92	*F* = 198.62	*F* = 0.20	*F* = 14.47		*F* = 41.67		
	***p*** = **2.64 × 10^−15^**	***p*** = **2.64 × 10^−15^**	*p* = 0.7154	***p*** = **2.64 × 10^−15^**		***p*** = **2.64 × 10^−15^**		
lactate	*F* = 0.50	*F* = 0.21	*F* = 2.12	*F* = 23.29				
	*p* = 0.5561	*p* = 0.7055	*p* = 0.2025	***p*** = **2.64 × 10^−15^**				
pyruvate	*F* = 1.72	*F* = 3.73	*F* = 0.35	*F* = 16.27	*F* = 6.22			
	*p* = 0.2559	*p* = 0.0850	*p* = 0.6244	***p*** = **2.64 × 10^−15^**	***p*** = **0.0009**			
acetate	*F* = 52.88	*F* = 38.72	*F* = 0.40	*F* = 7.19		*F* = 9.63		
	***p*** = **4.33 × 10^−12^**	***p*** = **3.64 × 10^−9^**	*p* = 0.6001	***p*** = **2.64 × 10^−15^**		***p*** = **9.61 × 10^−6^**		
**glycoprotein acetyls (GlycA)**								
GlycA	*F* = 37.27	*F* = 128.19	*F* = 11.82	*F* = 32.29	*F* = 8.69	*F* = 1.48	*F* = 3.15	
	***p*** = **7.29 × 10^−9^**	***p*** = **2.64 × 10^−15^**	***p*** = **0.0015**	***p*** = **2.64 × 10^−15^**	***p*** = **3.35 × 10^−5^**	*p* = 0.287	*p* = 0.116	

#### 3.2.3. Glycolysis

Breed affected all glycolysis-related measurands, but there was no clear pattern in the breed differences (electronic supplementary material, figure S4, table S4, data S2). Age influenced citrate, glucose and acetate levels (table 2; electronic supplementary material, figure S12). Glucose levels were higher in puppies and senior dogs than in adult dogs (linear effect: *F* = 421.92, d.f. = 1, *p* = 2.643 × 10^−15^; quadratic effect: *F* = 198.62, d.f. = 1, *p* = 2.643 × 10^−15^). Females had higher citrate levels but there were no sex differences in other measurands (electronic supplementary material, figure S20, data S2). Diet affected citrate and pyruvate, as dogs eating raw food had higher levels of both measurands than dogs consuming other diet types (electronic supplementary material, figure S28, data S2). Fasting affected citrate, glucose and acetate (electronic supplementary material, figure S36, data S2). For example, glucose concentration was significantly higher in dogs that fasted less than 8 h than in dogs that fasted greater than or equal to 8 h.

#### 3.3.4. Inflammation

**Figure 3.**
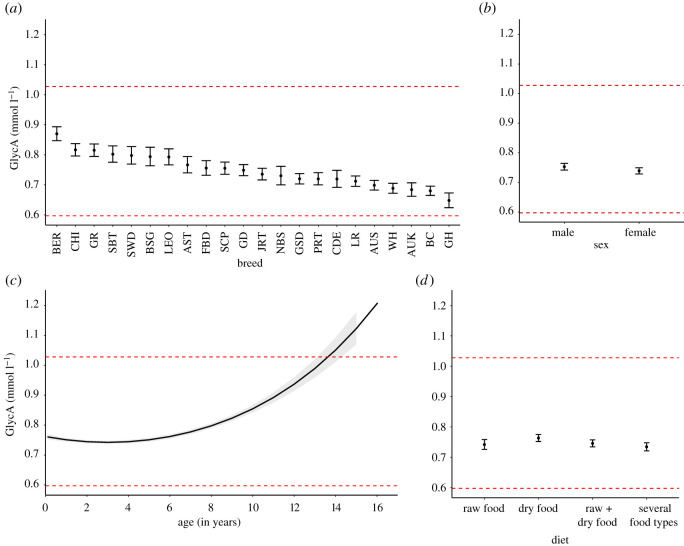
The significant influences on inflammation marker GlycA serum concentration. (*a*) GlycA concentration differed between breeds. (*b*) Males had higher GlycA concentration than females. (*c*) GlycA concentration increased with age, and dogs over 14 years old had GlycA levels above the upper RL. (*d*) Dogs consuming dry food diet had higher GlycA concentration than dogs consuming other diet types. Red dashed lines indicate lower and upper reference limits of the NMR method, calculated for dogs of all ages [32]. Error bars (*a*,*b*,*d*) and grey lines (*c*) indicate 95% confidence limits. *N* = 2068. AST, American Staffordshire Terrier; AUK, Australian Kelpie; AUS, Australian Shepherd; BSG, Belgian Shepherd Dog Groenendael; BER, Bernese Mountain Dog; BC, Border Collie; CHI, Chihuahua (both coat types); CDE, Cirneco d'ell Etna; FBD, French Bulldog; GSD, German Shepherd Dog; GŖ Golden Retriever; GD, Great Dane; GH, Greyhound; JRT, Jack Russell Terrier; LR, Labrador Retriever, LEO, Leonberger; NBS, Norrbottenspitz; PRT, Parson Russell Terrier; SCP, Schapendoes; SWD, Spanish Waterdog; SBT, Staffordshire Bullterrier; WH, Whippet.

The highest glycoprotein acetyls (GlycA) concentrations were found in BER, CHI and GR, whereas GH had the lowest concentration, near the lower reference limit (figure 3; electronic supplementary material, table S4, data S2). Males had higher GlycA (*Z* = 3.44, d.f. = 1, *p* = 0.001) (figure 3; electronic supplementary material, data S2). After 8 years of age, GlycA concentration increased dramatically even exceeding the upper RL around 14 years of age (linear effect: *F* = 37.27, d.f. = 1, *p* = 7.293 × 10^−9^; quadratic effect: *F* = 128.19, d.f. = 1, *p* = 2.643 × 10^−15^) (figure 3 and table 2). Dogs eating dry food had significantly higher GlycA levels than dogs consuming other diet types (figure 3; electronic supplementary material, data S2).

#### 3.2.5. Cholesterol

Breed affected all cholesterol measurands with clear pattern as muscular and slender breeds GH, CHI, WH and PRT had the lowest and heavy-structured breeds BER and GR had the highest concentrations (electronic supplementary material, figure S6, table S4, data S2). All measurands increased with age, the levels elevating more rapidly after 8 years of age (electronic supplementary material, figure S14; table 3). Females had higher levels of all other measurands except VLDL cholesterol, which showed no sex difference (electronic supplementary material, figure S22, data S2). LDL cholesterol was not influenced by diet, but in all other measurands, dogs eating raw food had lower levels than dogs eating dry food (electronic supplementary material, figure S30, data S2). Fasting explained variation only in VLDL cholesterol (electronic supplementary material, figure S38, data S1).

**Table 3.** Variables best explaining variation in cholesterol and triglyceride concentrations in dogs. *p*-values were controlled for false discovery rate (FDR). Significant effects are indicated in bold (FDR *p* < 0.05). *N* = 2068. The full names of the measurands are reported in electronic supplementary material, table S1.

variable	age	age^2^	sex	breed	diet	fasting
	d.f. = 1	d.f. = 1	d.f. = 1	d.f. = 21	d.f. = 3	d.f. = 3
**cholesterol**						
total cholesterol	*F* = 2.71	*F* = 12.79	*F* = 53.82	*F* = 39.36	*F* = 2.82	
	*p* = 0.1465	***p*** = **0.0009**	***p*** = **2.79 × 10^−12^**	***p*** = **2.64 × 10^−15^**	*p* = 0.0617	
est. cholesterol	*F* = 1.49	*F* = 9.93	*F* = 58.85	*F* = 37.76	*F* = 2.99	
	*p* = 0.2924	***p*** = **0.0037**	***p*** = **2.60 × 10^−13^**	***p*** = **2.64 × 10^−15^**	*p* = 0.0508	
free cholesterol	*F* = 9.43	*F* = 24.65	*F* = 37.12	*F* = 43.93	*F* = 2.28	
	***p*** = **0.0048**	***p*** = **3.00 × 10^−6^**	***p*** = **7.82 × 10^−9^**	***p*** = **2.64 × 10^−15^**	*p* = 0.1171	
HDL cholesterol	*F* = 2.15	*F* = 7.99	*F* = 75.33	*F* = 31.90	*F* = 3.52	
	*p* = 0.1989	***p*** = **0.0097**	***p*** = **2.64 × 10^−15^**	***p*** = **2.64 × 10^−15^**	***p*** = **0.0266**	
LDL cholesterol	*F* = 5.62	*F* = 20.89	*F* = 7.87	*F* = 46.01		
	***p*** = **0.0321**	***p*** = **1.83 × 10^−5^**	***p*** = **0.0103**	***p*** = **2.64 × 10^−15^**		
VLDL cholesterol	*F* = 270.49		*F* = 0.10	*F* = 30.95	*F* = 5.64	*F* = 8.21
	***p*** = **2.64 × 10^−15^**		*p* = 0.7967	***p*** = **2.64 × 10^−15^**	***p*** = **0.0018**	***p*** = **6.35 × 10^−5^**
**triglycerides**						
HDL triglycerides	*F* = 184.72	*F* = 41.50	*F* = 1.50	*F* = 9.90	*F* = 7.98	
	***p*** = **2.64 × 10^−15^**	***p*** = **9.62 × 10^−10^**	*p* = 0.2903	***p*** = **2.64 × 10^−15^**	***p*** = **8.63 × 10^−5^**	
LDL triglycerides	*F* = 291.51	*F* = 188.94	*F* = 15.34	*F* = 18.68	*F* = 4.75	*F* = 10.24
	***p*** = **2.64 × 10^−15^**	***p*** = **2.64 × 10^−15^**	***p*** = **0.0003**	***p*** = **2.64 × 10^−15^**	***p*** = **0.0057**	***p*** = **4.23 × 10^−6^**
total triglycerides	*F* = 6.37	*F* = 51.15	*F* = 0.49	*F* = 17.96	*F* = 13.63	*F* = 6.90
	***p*** = **0.0219**	***p*** = **9.89 × 10^−12^**	*p* = 0.5588	***p*** = **2.64 × 10^−15^**	***p*** = **4.46 × 10^−8^**	***p*** = **0.0004**

#### 3.2.6. Triglycerides

Breed affected triglycerides with similar patterns seen in cholesterol measurands (electronic supplementary material, figure S7, table S4, data S2). Age had differential effects on the three triglyceride measurands (electronic supplementary material, figure S15; table 3). Females had higher LDL triglycerides, but sex differences were not observed in HDL and total triglycerides (electronic supplementary material, figure S23, data S2). Dogs eating raw food had lower triglyceride levels than dogs consuming other diet types (electronic supplementary material, figure S31, data S2). Fasting time did not influence HDL triglycerides, but shorter fasting times were associated with higher total triglycerides and LDL triglycerides (electronic supplementary material, figure S39, data S2).

#### 3.2.7. Fatty acids

Most fatty acid measurand levels were low in slender and muscular breeds (GH, WH, CHI, JRT, PRT), whereas heavy-structured breeds (BER, LEO, GR) had systematically high levels (electronic supplementary material, figure S8, table S4, data S2). However, GH had the highest levels of OleA%, PalA%, SFA% and Omega-6/Omega-3 fatty acids. Age significantly affected all other measurands than DPA and Omega-6%, but the age effects were not uniform (electronic supplementary material, figure S16; table 4). The total concentration of fatty acids increased with age nonlinearly (linear effect: *F* = 3.03, d.f. = 1, *p* = 0.124; quadratic effect: *F* = 25.68, d.f. = 1, FDR *p* = 1.830 × 10^−6^). The levels of most measurands, including total fatty acids, were higher in females and DPA%, Omega-6%, PalA%, PUFA%, SFA% and SteA% were influenced by the interaction between sex and sterilization (electronic supplementary material, figure S24, data S2). Most measurands were higher in dogs eating dry food or raw + dry food than in dogs eating solely raw food, except PalA%, SFA%, SteA% and Omega-6/Omega-3, which were highest in dogs eating solely raw food (electronic supplementary material, figure S32, data S1). Fasting time influenced most measurands, and commonly, shorter fasting times were associated with higher measurand levels (electronic supplementary material, figure S40, data S1).

**Table 4.** Variables best explaining variation in fatty acid measurand levels in dogs. *p*-values were controlled for false discovery rate (FDR). Significant effects are indicated in bold (FDR *p* < 0.05). *N* = 2068. ster, sterilization; sex*ster, interaction of sex and sterilization. The full names of the measurands are reported in electronic supplementary material, table S1.

variable	age	age^2^	sex	breed	diet	fasting	ster	sex*ster
	d.f. = 1	d.f. = 1	d.f. = 1	d.f. = 21	d.f. = 3	d.f. = 3	d.f. = 1	d.f. = 1
AA	*F* = 32.95	*F* = 50.97	*F* = 90.01	*F* = 26.44	*F* = 2.84			
	***p*** = **5.69 × 10^−8^**	***p*** = **1.07 × 10^−11^**	***p*** = **2.64 × 10^−15^**	***p*** = **2.64 × 10^−15^**	*p* = 0.0608			
AA %	*F* = 78.40	*F* = 43.25	*F* = 66.89	*F* = 6.42		*F* = 13.02		
	***p*** = **2.64 × 10^−15^**	***p*** = **4.18 × 10^−10^**	***p*** = **5.82 × 10^−15^**	***p*** = **2.64 × 10^−15^**		***p*** = **1.01 × 10^−7^**		
DHA	*F* = 157.31	*F* = 129.54	*F* = 80.37	*F* = 10.38	*F* = 7.85	*F* = 4.32	*F* = 10.82	
	***p*** = **2.64 × 10^−15^**	***p*** = **2.64 × 10^−15^**	***p*** = **2.64 × 10^−15^**	***p*** = **2.64 × 10^−15^**	***p*** = **0.0001**	***p*** = **0.0098**	***p*** = **0.0024**	
DHA %	*F* = 161.42	*F* = 103.59	*F* = 40.90	*F* = 6.24	*F* = 4.86	*F* = 2.89	*F* = 14.77	
	***p*** = **2.64 × 10^−15^**	***p*** = **2.64 × 10^−15^**	***p*** = **1.28 × 10^−9^**	***p*** = **2.64 × 10^−15^**	***p*** = **0.0050**	*p* = 0.0575	***p*** = **0.0003**	
DPA	*F* = 4.59	*F* = 2.03	*F* = 133.86	*F* = 23.16	*F* = 8.26			
	*p* = 0.0542	*p* = 0.2134	***p*** = **2.64 × 10^−15^**	***p*** = **2.64 × 10^−15^**	***p*** = **5.97 × 10^−5^**			
DPA %	*F* = 20.31	*F* = 12.05	*F* = 0.50	*F* = 4.70	*F* = 3.30	*F* = 13.16	*F* = 21.40	*F* = 7.70
	***p*** = **2.42 × 10^−5^**	***p*** = **0.0013**	*p* = 0.5547	***p*** = **7.60 × 10^−11^**	***p*** = **0.0347**	***p*** = **8.47 × 10^−8^**	***p*** = **1.43 × 10^−5^**	***p*** = **0.0112**
LA	*F* = 141.30		*F* = 32.89	*F* = 42.25	*F* = 10.62			
	***p*** = **2.64 × 10^−15^**		***p*** = **5.86 × 10^−8^**	***p*** = **2.64 × 10^−15^**	***p*** = **2.58 × 10^−6^**			
LA %	*F* = 292.05	*F* = 172.44	*F* = 22.65	*F* = 13.33	*F* = 7.20	*F* = 13.87		
	***p*** = **2.64 × 10^−15^**	***p*** = **2.64 × 10^−15^**	***p*** = **7.84 × 10^−6^**	***p*** = **2.64 × 10^−15^**	***p*** = **0.0002**	***p*** = **3.20 × 10^−8^**		
OleA	*F* = 173.16		*F* = 20.17	*F* = 31.25	*F* = 11.13	*F* = 11.68		
	***p*** = **2.64 × 10^−15^**		***p*** = **2.59 × 10^−5^**	***p*** = **2.64 × 10^−15^**	***p*** = **1.30 × 10^−6^**	***p*** = **6.20 × 10^−7^**		
OleA %	*F* = 100.50	*F* = 56.98	*F* = 26.26	*F* = 11.32		*F* = 5.65		
	***p*** = **2.64 × 10^−15^**	***p*** = **6.29 × 10^−13^**	***p*** = **1.39 × 10^−6^**	***p*** = **2.64 10^−15^**		***p*** = **0.0018**		
Omega-3	*F* = 49.37	*F* = 61.08	*F* = 72.36	*F* = 15.86	*F* = 10.16	*F* = 5.50	*F* = 11.62	
	***p*** = **2.30 × 10^−11^**	***p*** = **9.08 × 10^−14^**	***p*** = **2.64 × 10^−15^**	***p*** = **2.64 × 10^−15^**	***p*** = **4.79 × 10^−6^**	***p*** = **0.0022**	***p*** = **0.0016**	
Omega-3%	*F* = 46.26	*F* = 32.05	*F* = 24.54	*F* = 5.88	*F* = 5.98	*F* = 3.37	*F* = 17.37	
	***p*** = **1.01 × 10^−10^**	***p*** = **8.77 × 10^−8^**	***p*** = **3.15 × 10^−6^**	***p*** = **6.80 × 10^−15^**	***p*** = **0.0012**	***p*** = **0.0321**	***p*** = **9.98 × 10^−5^**	
Omega-6	*F* = 3.16	*F* = 22.64	*F* = 52.50	*F* = 38.83	*F* = 6.20	*F* = 2.07		
	*p* = 0.1150	***p*** = **7.86 × 10^−6^**	***p*** = **5.20 × 10^−12^**	***p*** = **2.64 × 10^−15^**	***p*** = **0.0009**	*p* = 0.1495		
Omega-6%	*F* = 0.98	*F* = 1.75	*F* = 4.72	*F* = 14.67		*F* = 27.22	*F* = 3.05	*F* = 15.73
	*p* = 0.4010	*p* = 0.2502	*p* = 0.0507	***p*** = **2.64 × 10^−15^**		***p*** = **2.64 × 10^−15^**	*p* = 0.1220	***p*** = **0.0002**
PalA	*F* = 3.58	*F* = 26.95	*F* = 25.51	*F* = 36.56	*F* = 5.40	*F* = 13.17		
	*p* = 0.0920	***p*** = **1.00 × 10^−6^**	***p*** = **1.99 × 10^−6^**	***p*** = **2.64 × 10^−15^**	***p*** = **0.0025**	***p*** = **8.31 × 10^−8^**		
PalA %	*F* = 1.33	*F* = 5.43	*F* = 1.56	*F* = 17.30	*F* = 11.07	*F* = 11.93	*F* = 32.09	*F* = 26.78
	*p* = 0.3210	***p*** = **0.0352**	*p* = 0.2799	***p*** = **2.64 × 10^−15^**	***p*** = **1.40 × 10^−6^**	***p*** = **4.40 × 10^−7^**	***p*** = **8.63 × 10^−8^**	***p*** = **1.08 × 10^−6^**
PUFA	*F* = 9.15	*F* = 35.66	*F* = 70.53	*F* = 40.64	*F* = 8.13	*F* = 2.78		
	***p*** = **0.0055**	***p*** = **1.56 × 10^−8^**	***p*** = **2.64 × 10^−15^**	***p*** = **2.64 × 10^−15^**	***p*** = **7.09 × 10^−5^**	*p* = 0.0653		
PUFA %	*F* = 65.71	*F* = 52.43	*F* = 1.49	*F* = 15.86	*F* = 4.55	*F* = 8.48	*F* = 27.08	*F* = 21.68
	***p*** = **1.01 × 10^−14^**	***p*** = **5.37 × 10^−12^**	*p* = 0.2927	***p*** = **2.64 × 10^−15^**	***p*** = **0.0073**	***p*** = **4.48 × 10^−5^**	***p*** = **9.40 × 10^−7^**	***p*** = **1.25 × 10^−5^**
SFA	*F* = 2.11	*F* = 22.06	*F* = 36.01	*F* = 36.34	*F* = 6.07	*F* = 8.99		
	*p* = 0.2039	***p*** = **1.04 × 10^−5^**	***p*** = **1.32 × 10^−8^**	***p*** = **2.64 × 10^−15^**	***p*** = **0.0011**	***p*** = **2.27 × 10^−5^**		
SFA %	*F* = 12.01	*F* = 18.49	*F* = 0.39	*F* = 18.32	*F* = 19.49	*F* = 14.25	*F* = 34.78	*F* = 10.63
	***p*** = **0.0013**	***p*** = **5.82 × 10^−5^**	*p* = 0.6046	***p*** = **2.64 × 10^−15^**	***p*** = **1.51 × 10^−11^**	***p*** = **1.91 × 10^−8^**	***p*** = **2.37 × 10^−8^**	***p*** = **0.0026**
SteA	*F* = 1.15	*F* = 17.61	*F* = 44.17	*F* = 34.99	*F* = 6.38	*F* = 6.08		
	*p* = 0.3594	***p*** = **8.89 × 10^−5^**	***p*** = **2.71 × 10^−10^**	***p*** = **2.64 × 10^−15^**	***p*** = **0.0007**	***p*** = **0.0010**		
SteA %	*F* = 18.25	*F* = 14.66	*F* = 9.99	*F* = 12.13	*F* = 7.35	*F* = 6.88	*F* = 1.46	*F* = 7.20
	***p*** = **6.53 × 10^−5^**	***p*** = **0.0004**	***p*** = **0.0036**	***p*** = **2.64 × 10^−15^**	***p*** = **0.0002**	***p*** = **0.0004**	*p* = 0.2985	***p*** = **0.0144**
total FA	*F* = 3.03	*F* = 25.68	*F* = 52.87	*F* = 39.62	*F* = 7.99	*F* = 5.61		
	*p* = 0.1235	***p*** = **1.83 × 10^−6^**	***p*** = **4.36 × 10^−12^**	***p*** = **2.64 × 10^−15^**	***p*** = **8.48 × 10^−5^**	***p*** = **0.0019**		
Omega-6/omega-3	*F* = 53.33	*F* = 35.41	*F* = 21.53	*F* = 5.36	*F* = 3.53	*F* = 4.51	*F* = 16.94	
	***p*** = **3.51 × 10^−12^**	***p*** = **1.76 × 10^−8^**	***p*** = **1.34 × 10^−5^**	***p*** = **4.34 × 10^−13^**	***p*** = **0.0264**	***p*** = **0.0077**	***p*** = **0.0001**	

#### 3.2.8. Lipoproteins

For most lipoprotein measurands, BER and GR had the highest and GH and CHI the lowest concentrations (electronic supplementary material, figure S9, table S4, data S2). However, breeds that otherwise had low concentrations of almost all other lipoproteins (GH, CHI, WH, PRT, JRT) had the largest diameter of LDL particles, and BER, GR and AST had the smallest diameter of LDL particles. Age affected all other measurands except HDL particle size and most XL-HDL measurands (electronic supplementary material, figure S17; table 5). The levels of most measurands increased with age, and L-LDL-L, L-LDL-FC, L-LDL-PL, VLDL particles, VLDL lipids, all S-VLDL measurands, XL-VLDL particles and XL-VLDL-C even exceeded the upper RL after 14 years of age. Females had higher levels of most HDL and LDL measurands (electronic supplementary material, figure S25, data S2). Sex differences were observed in multiple VLDL measurands, but clear patterns were absent. Sterilization affected only one lipoprotein measurand: neutered dogs had lower levels of S-HDL-TG than intact dogs (*Z* = −2.53, d.f. = 1, *p* = 0.021). Diet affected most HDL and VLDL measurands but only a few LDL measurands (electronic supplementary material, figure S33, data S2). In most measurands, dogs eating dry food had the highest and dogs eating raw food the lowest levels. There were no clear patterns in fasting time and measurand concentrations (electronic supplementary material, figure S41, data S2).

**Table 5.** Variables best explaining variation in lipoprotein measurand levels in dogs. *p*-values were controlled for false discovery rate (FDR). Significant effects are indicated in bold (FDR *p* < 0.05). *N* = 2068. The full names of the measurands are reported in electronic supplementary material, table S1.

variable	age	age^2^	sex	breed	diet	fasting	sterilization
	d.f. = 1	d.f. = 1	d.f. = 1	d.f. = 21	d.f. = 3	d.f. = 3	d.f. = 1
**lipoprotein particle size**							
HDL diameter	*F* = 0.02	*F* = 2.47	*F* = 2.68	*F* = = 42.21			
	*p* = 0.9207	*p* = 0.1668	*p* = 0.15	***p*** = **2.64 × 10^−15^**			
LDL diameter	*F* = 120.67	*F* = 54.58	*F* = 44.73	*F* = 18.36		*F* = 12.90	
	***p*** = **2.64 × 10^−15^**	***p*** = **1.96 × 10^−12^**	***p*** = **2.06 × 10^−10^**	***p*** = **2.64 × 10^−15^**		***p*** = **1.20 × 10^−7^**	
VLDL diameter	*F* = 187.60	*F* = 46.01	*F* = 4.73	*F* = 13.48		*F* = 2.89	
	***p*** = **2.64 × 10^−15^**	***p*** = **1.13 × 10^−10^**	*p* = 0.0505	***p*** = = **2.64 × 10^−15^**		*p* = 0.0570	
**HDL lipoproteins**							
HDL lipids	*F* = 5.35	*F* = 14.50	*F* = 63.58	*F* = 30.95	*F* = 5.11	*F* = 3.03	
	***p*** = **0.0367**	***p*** = **0.0004**	***p*** = **2.77 × 10^−14^**	***p*** = **2.64 × 10^−15^**	***p*** = **0.0036**	***p*** = **0.0486**	
HDL particles	*F* = 12.32	*F* = 16.09	*F* = 114.73	*F* = 20.21	*F* = 7.50		
	***p*** = **0.0012**	***p*** = **0.0002**	***p*** = **2.64 × 10^−15^**	***p*** = **2.64 × 10^−15^**	***p*** = **0.0002**		
S-HDL	*F* = 2.86	*F* = 5.83	*F* = 117.34	*F* = 13.59	*F* = 10.37		
	*p* = 0.1347	***p*** = **0.0288**	***p*** = **2.64 × 10^−15^**	***p*** = **2.64 × 10^−15^**	***p*** =		
S-HDL-L	*F* = 16.31	*F* = 19.07	*F* = 101.14	*F* = 12.78	*F* = 13.98	*F* = 4.77	
	***p*** = **0.0002**	***p*** = **4.40 × 10^−5^**	***p*** = **2.64 × 10^−15^**	***p*** = **2.64 × 10^−15^**	***p*** = **2.77 × 10^−8^**	***p*** = **0.0056**	
S-HDL-C	*F* = 6.78	*F* = 8.06	*F* = 121.80	*F* = 13.28	*F* = 10.06		
	***p*** = **0.0178**	***p*** = **0.0094**	***p*** = **2.64 × 10^−15^**	***p*** = **2.64 × 10^−15^**	***p*** = **5.41 × 10^−6^**		
S-HDL-CE	*F* = 6.88	*F* = 6.11	*F* = 127.19	*F* = 11.61	*F* = 9.69		
	***p*** = **0.0170**	***p*** = **0.0250**	***p*** = **2.64 × 10^−15^**	***p*** = **2.64 × 10^−15^**	***p*** = **8.88 × 10^−6^**		
S-HDL-FC	*F* = 1.80	*F* = 9.81	*F* = 75.90	*F* = 21.68	*F* = 9.92	*F* = 4.87	
	*p* = 0.2430	***p*** = **0.0040**	***p*** = **2.64 × 10^−15^**	***p*** = **2.64 × 10^−15^**	***p*** = **6.53 × 10^−6^**	***p*** = **0.0049**	
S-HDL-PL	*F* = 32.90	*F* = 33.18	*F* = 76.95	*F* = 12.03	*F* = 16.54	*F* = 16.50	
	***p*** = **5.84 × 10^−8^**	***p*** = **5.10 × 10^−8^**	***p*** = **2.64 × 10^−15^**	***p*** = **2.64 × 10^−15^**	***p*** = **8.45 × 10^−10^**	***p*** = **9.02 × 10^−10^**	
S-HDL-TG	*F* = 167.35	*F* = 40.84	*F* = 5.70	*F* = 6.91	*F* = 3.97	*F* = 4.36	*F* = 6.29
	***p*** = **2.64 × 10^−15^**	***p*** = **1.32 × 10^−9^**	***p*** = **0.0308**	***p*** = **2.64 × 10^−15^**	***p*** = **0.0153**	***p*** = **0.0093**	***p*** = **0.0229**
L-HDL	*F* = 26.28	*F* = 24.52	*F* = 126.05	*F* = 16.51	*F* = 9.42	*F* = 2.30	
	***p*** = **1.37 × 10^−6^**	***p*** = **3.18 × 10^−6^**	***p*** = **2.64 × 10^−15^**	***p*** = **2.64 × 10^−15^**	***p*** = **1.28 × 10^−5^**	*p* = 0.1148	
L-HDL-L	*F* = 23.67	*F* = 30.01	*F* = 80.42	*F* = 17.51	*F* = 11.64	*F* = 13.54	
	***p*** = **4.79 × 10^−6^**	***p*** = **2.33 × 10^−7^**	***p*** = **2.64 × 10^−15^**	***p*** = **2.64 × 10^−15^**	***p*** = **6.51 × 10^−7^**	***p*** = **5.00 × 10^−8^**	
L-HDL-C	*F* = 11.76	*F* = 15.44	*F* = 108.38	*F* = 20.47	*F* = 7.60	*F* = 2.33	
	***p*** = **0.0015**	***p*** = **0.0003**	***p*** = **2.64 × 10^−15^**	***p*** = **2.64 × 10^−15^**	***p*** = **0.0001**	*p* = 0.1104	
L-HDL-CE	*F* = 10.96	*F* = 14.35	*F* = 111.66	*F* = 19.55	*F* = 7.38		
	***p*** = **0.0022**	***p*** = **0.0004**	***p*** = **2.64 × 10^−15^**	***p*** = **2.64 × 10^−15^**	***p =* 0.0002**		
L-HDL-FC	*F* = 30.93	*F* = 34.63	*F* = 79.04	*F* = 25.22	*F* = 7.20	*F* = 6.62	
	***p*** = **1.49 × 10^−7^**	***p*** = **2.55 × 10^−8^**	***p*** = **2.64 × 10^−15^**	***p*** = **2.64 × 10^−15^**	***p*** = **0.0002**	***p*** = **0.0005**	
L-HDL-PL	*F* = 39.78	*F* = 46.90	*F* = 31.79	*F* = 12.11	*F* = 14.33	*F* = 42.32	
	***p*** = **2.20 × 10^−9^**	***p*** = **7.39 × 10^−11^**	***p*** = **9.93 × 10^−8^**	***p*** = **2.64 × 10^−15^**	***p*** = **1.72 × 10^−8^**	***p*** = **2.64 × 10^−15^**	
L-HDL-TG	*F* = 158.22	*F* = 31.54	*F* = 0.35	*F* = 10.07	*F* = 9.02	*F* = 7.64	
	***p*** = **2.64 × 10^−15^**	***p*** = **1.12 × 10^−7^**	*p* = 0.6261	***p*** = **2.64 × 10^−15^**	***p*** = **2.18 × 10^−5^**	***p*** = **0.0001**	
XL-HDL	*F* = 0.63	*F* = 1.45	*F* = 19.43	*F* = 41.17			
	*p* = 0.5053	*p* = 0.2990	***p*** = **3.69 × 10^−5^**	***p*** = **2.64 × 10^−15^**			
XL-HDL-L	*F* = 0.18	*F* = 2.66	*F* = 22.71	*F* = 42.61			
	*p* = 0.7294	*p* = 0.1500	***p*** = **7.64 × 10^−6^**	***p*** = **2.64 × 10^−15^**			
XL-HDL-C	*F* = 0.38	*F* = 1.81	*F* = 26.54	*F* = 41.87			
	*p* = 0.6077	*p* = 0.2421	***p*** = **1.21 × 10^−6^**	***p*** = **2.64 × 10^−15^**			
XL-HDL-CE	*F* = 0.69	*F* = 1.40	*F* = 28.13	*F* = 41.44			
	*p* = 0.4839	*p* = 0.3090	***p*** = **5.68 × 10^−7^**	***p*** = **2.64 × 10^−15^**			
XL-HDL-FC	*F* = 0.02	*F* = 3.64	*F* = 21.03	*F* = 42.97			
	*p* = 0.9178	*p* = 0.0891	***p*** = **1.71 × 10^−5^**	***p*** = **2.64 × 10^−15^**			
XL-HDL-PL	*F* = 0.01	*F* = 3.99	*F* = 17.54	*F* = 42.27			
	*p* = 0.9489	*p* = 0.0741	***p*** = **9.17 × 10^−5^**	***p*** = **2.64 × 10^−15^**			
XL-HDL-TG	*F* = 50.37	*F* = 0.93	*F* = 0.74	*F* = 15.86	*F* = 10.70		
	***p*** = **1.43 × 10^−11^**	*p* = 0.4134	*p* = 0.4682	***p*** = **2.64 × 10^−15^**	***p*** = **2.30 × 10^−6^**		
**LDL lipoproteins**							
LDL lipids	*F* = 19.03	*F* = 36.91	*F* = 9.47	*F* = 47.04			
	***p*** = **4.50 × 10^−5^**	***p*** = **8.62 × 10^−9^**	***p*** = **0.0047**	***p*** = **2.64 × 10^−15^**			
LDL particles	*F* = 10.58	*F* = 26.65	*F* = 13.84	*F* = 46.60			
	***p*** = **0.0027**	***p*** = **1.15 × 10^−6^**	***p*** = **0.0005**	***p*** = **2.64 × 10^−15^**			
S-LDL	*F* = 0.88	*F* = 10.30	*F* = 20.99	*F* = 43.84		*F* = 3.72	
	*p* = 0.4283	***p*** = **0.0031**	***p*** = **1.74 × 10^−5^**	***p*** = **2.64 × 10^−15^**		***p*** = **0.0206**	
S-LDL-L	*F* = 1.01	*F* = 10.36	*F* = 17.30	*F* = 44.17			
	*p* = 0.3931	***p*** = **0.0031**	***p*** = **0.0001**	***p*** = **2.64 × 10^−15^**			
S-LDL-C	*F* = 1.38	*F* = 11.10	*F* = 16.01	*F* = 43.28		*F* = 3.65	
	*p* = 0.3119	***p*** = **0.0021**	***p*** = **0.0002**	***p*** = **2.64 × 10^−15^**		***p*** = **0.0228**	
S-LDL-CE	*F* = 0.90	*F* = 10.94	*F* = 15.27	*F* = 43.40		*F* = 3.30	
	*p* = 0.4218	***p*** = **0.0023**	***p*** = **0.0003**	***p*** = **2.64 × 10^−15^**		***p*** = **0.0349**	
S-LDL-FC	*F* = 2.91	*F* = 11.09	*F* = 17.59	*F* = 42.34		*F* = 4.50	
	*p* = 0.1313	***p*** = **0.0021**	***p*** = **8.96 × 10^−5^**	***p*** = **2.64 × 10^−15^**		***p*** = **0.0078**	
S-LDL-PL	*F* = 0.98	*F* = 10.27	*F* = 17.57	*F* = 44.80			
	*p* = 0.4016	***p*** = **0.0032**	***p*** = **9.07 × 10^−5^**	***p*** = **2.64 × 10^−15^**			
S-LDL-TG	*F* = 108.10	*F* = 87.71	*F* = 31.00	*F* = 24.50	*F* = 3.65		
	***p*** = **2.64 × 10^−15^**	***p*** = **2.64 × 10^−15^**	***p*** = **1.45 × 10^−7^**	***p*** = **2.64 × 10^−15^**	***p*** = **0.0228**		
L-LDL	*F* = 122.24	*F* = 126.66	*F* = 0.30	*F* = 42.46			
	***p*** = **2.64 × 10^−15^**	***p*** = **2.64 × 10^−15^**	*p* = 0.6524	***p*** = **2.64 × 10^−15^**			
L-LDL-L	*F* = 126.67	*F* = 136.61	*F* = 0.01	*F* = 42.41		*F* = 1.03	
	***p*** = **2.64 × 10^−15^**	***p*** = **2.64 × 10^−15^**	*p* = 0.9305	***p*** = **2.64 × 10^−15^**		*p* = 0.4589	
L-LDL-C	*F* = 39.97	*F* = 64.63	*F* = 0.40	*F* = 41.90			
	***p*** = **2.00 × 10^−9^**	***p*** = **1.68 × 10^−14^**	*p* = 0.6004	***p*** = **2.64 × 10^−15^**			
L-LDL-CE	*F* = 18.16	*F* = 43.37	*F* = 0.57	*F* = 40.77			
	***p*** = **6.81 × 10^−5^**	***p*** = **3.96 × 10^−10^**	*p* = 0.5282	***p*** = **2.64 × 10^−15^**			
L-LDL-FC	*F* = 134.58	*F* = 129.13	*F* = 0.02	*F* = 39.96			
	***p*** = **2.64 × 10^−15^**	***p*** = **2.64 × 10^−15^**	*p* = 0.9123	***p*** = **2.64 × 10^−15^**			
L-LDL-PL	*F* = 156.02	*F* = 149.28	*F* = 0.33	*F* = 34.68		*F* = 1.58	
	***p*** = **2.64 × 10^−15^**	***p*** = **2.64 × 10^−15^**	*p* = 0.6320	***p*** = **2.64 × 10^−15^**		*p* = 0.2591	
L-LDL-TG	*F* = 329.09	*F* = 201.78	*F* = 9.52	*F* = 15.42	*F* = 4.60	*F* = 17.12	
	***p*** = **2.64 × 10^−15^**	***p*** = **2.64 × 10^−15^**	***p*** = **0.0046**	***p*** = **2.64 × 10^−15^**	***p*** = **0.0069**	***p*** = **3.82 × 10^−10^**	
**VLDL lipoproteins**							
VLDL lipids	*F* = 0.0002	*F* = 29.72	*F* = 0.13	*F* = 21.64	*F* = 10.71	*F* = 9.68	
	*p* = 0.9919	***p*** = **2.67 × 10^−7^**	*p* = 0.7679	***p*** = **2.64 × 10^−15^**	***p*** = **2.28 × 10^−6^**	***p*** = **9.05 × 10^−6^**	
VLDL particles	*F* = 40.33	*F* = 101.34	*F* = 4.38	*F* = 32.48	*F* = 7.50	*F* = 14.28	
	***p*** = **1.68 × 10^−9^**	***p*** = **2.64 × 10^−15^**	*p* = 0.0606	***p*** = **2.64 × 10^−15^**	***p*** = **0.0002**	***p*** = **1.83 × 10^−8^**	
S-VLDL	*F* = 136.99	*F* = 173.95	*F* = 1.85	*F* = 31.62	*F* = 6.12	*F* = 16.76	
	***p*** = **2.64 × 10^−15^**	***p*** = **2.64 × 10^−15^**	*p* = 0.2362	***p*** = **2.64 × 10^−15^**	***p*** = **0.0010**	***p*** = **6.30 × 10^−10^**	
S-VLDL-L	*F* = 69.31	*F* = 127.23	*F* = 0.53	*F* = 31.96	*F* = 9.30	*F* = 11.53	
	***p*** = **2.64 × 10^−15^**	***p*** = **2.64 × 10^−15^**	*p* = 0.5423	***p*** = **2.64 × 10^−15^**	***p*** = **1.50 × 10^−5^**	***p*** = **7.58 × 10^−7^**	
S-VLDL-C	*F* = 47.22	*F* = 73.69	*F* = 2.42	*F* = 29.10	*F* = 3.57	*F* = 2.80	
	***p*** = **6.34 × 10^−11^**	***p*** = **2.64 × 10^−15^**	*p* = 0.1717	***p*** = **2.64 × 10^−15^**	***p*** = **0.0253**	*p* = 0.0638	
S-VLDL-CE	*F* = 35.17	*F* = 50.79	*F* = 10.93	*F* = 23.76			
	***p*** = **1.97 × 10^−8^**	***p*** = **1.17 × 10^−11^**	***p*** = **0.0023**	***p*** = **2.64 × 10^−15^**			
S-VLDL-FC	*F* = 59.90	*F* = 100.28	*F* = 7.14	*F* = 32.21	*F* = 8.43	*F* = 5.74	
	***p*** = **1.58 × 10^−13^**	***p*** = **2.64 × 10^−15^**	***p*** = **0.0149**	***p*** = **2.64 × 10^−15^**	***p*** = **4.76 × 10^−5^**	***p*** = **0.0016**	
S-VLDL-PL	*F* = 118.10	*F* = 123.30	*F* = 34.16	*F* = 25.20	*F* = 2.45	*F* = 16.86	
	***p*** = **2.64 × 10^−15^**	***p*** = **2.64 × 10^−15^**	***p*** = **3.19 × 10^−8^**	***p*** = **2.64 × 10^−15^**	*p* = 0.0968	***p*** = **5.49 × 10^−10^**	
S-VLDL-TG	*F* = 15.85	*F* = 58.43	*F* = 5.66	*F* = 17.68	*F* = 10.46	*F* = 9.56	
	***p*** = **0.0002**	***p*** = **3.18 × 10^−13^**	***p*** = **0.0314**	***p*** = **2.64 × 10^−15^**	***p*** = **3.16 × 10^−6^**	***p*** = **1.05 × 10^−5^**	
L-VLDL	*F* = 85.86	*F* = 8.50	*F* = 7.56	*F* = 20.62	*F* = 4.90	*F* = 3.60	
	***p*** = **2.64 × 10^−15^**	***p*** = **0.0075**	***p*** = **0.0121**	***p*** = **2.64 × 10^−15^**	***p*** = **0.0047**	***p*** = **0.0244**	
L-VLDL-L	*F* = 397.26		*F* = 0.24	*F* = 19.24	*F* = 10.34	*F* = 4.64	
	***p*** = **2.64 × 10^−15^**		*p* = 0.6899	***p*** = **2.64 × 10^−15^**	***p*** = **3.73 × 10^−6^**	***p*** = **0.0066**	
L-VLDL-C	*F* = 106.72	*F* = 8.94	*F* = 0.54	*F* = 28.12	*F* = 5.29	*F* = 6.35	
	***p*** = **2.64 × 10^−15^**	***p*** = **0.0061**	*p* = 0.5403	***p*** = **2.64 × 10^−15^**	***p*** = **0.0029**	***p*** = **0.0007**	
L-VLDL-CE	*F* = 118.48	*F* = 12.67	*F* = 8.46	*F* = 32.56	*F* = 6.55	*F* = 8.72	
	***p*** = **2.64 × 10^−15^**	***p*** = **0.0010**	***p*** = **0.0077**	***p*** = **2.64 × 10^−15^**	***p*** = **0.0006**	***p*** = **3.22 × 10^−5^**	
L-VLDL-FC	*F* = 66.18	*F* = 2.66	*F* = 8.18	*F* = 20.97	*F* = 5.29	*F* = 7.70	
	***p*** = **8.13 × 10^−15^**	*p* = 0.1506	***p*** = **0.0088**	***p*** = **2.64 × 10^−15^**	***p*** = **0.0029**	***p*** = **0.0001**	
L-VLDL-PL	*F* = 180.77		*F* = 28.71	*F* = 15.43		*F* = 19.89	
	***p*** = **2.64 × 10^−15^**		***p*** = **4.31 × 10^−7^**	***p*** = **2.64 × 10^−15^**		***p*** = **8.71 × 10^−12^**	
L-VLDL-TG	*F* = 303.66		*F* = 2.64	*F* = 14.79	*F* = 13.74		
	***p*** = **2.64 × 10^−15^**		*p* = 0.1523	***p*** = **2.64 × 10^−15^**	***p*** = **3.85 × 10^−8^**		
XL-VLDL	*F* = 2.88	*F* = 4.94	*F* = 7.66	*F* = 8.74	*F* = 5.32	*F* = 13.80	
	*p* = 0.1339	***p*** = **0.0452**	***p*** = **0.0115**	***p*** = **2.64 × 10^−15^**	***p*** = **0.0028**	***p*** = **3.55 × 10^−8^**	
XL-VLDL-L	*F* = 194.93		*F* = 0.06	*F* = 9.73	*F* = 7.95	*F* = 14.39	
	***p*** = **2.64 × 10^−15^**		*p* = 0.8443	***p*** = **2.64 × 10^−15^**	***p*** = **9.01 × 10^−5^**	***p*** = **1.58 × 10^−8^**	
XL-VLDL-C	*F* = 1.59	*F* = 8.69	*F* = 10.33	*F* = 9.48	*F* = 4.25	*F* = 18.58	
	*p* = 0.2749	***p*** = **0.0069**	***p*** = **0.0031**	***p*** = **2.64 × 10^−15^**	***p*** = **0.0108**	***p*** = **5.27 × 10^−11^**	
XL-VLDL-TG	*F* = 174.98		*F* = 0.86	*F* = 9.10	*F* = 8.47	*F* = 8.15	
	***p*** = **2.64 × 10^−15^**		*p* = 0.4318	***p*** = **2.64 × 10^−15^**	*p* = **4.53 × 10^−5^**	*p* = **6.90 × 10^−5^**	

## 4. Discussion

Analyses in 2068 dogs from 22 different breeds indicated that the metabolism of healthy pet dogs is influenced by several physiological and diet-related factors, including breed, age, sex, sterilization, diet and fasting time before blood sampling. However, the effects were largely measurand/metabolite group dependent, but age and breed caused the most prominent changes in the measurand levels. These results indicate that the NMR metabolomics method is a valuable tool for metabolic studies in basic physiology and form a solid foundation for canine metabolomics studies examining disease associations.

Dog breeds differ in terms of genetics, morphology, physiology and behaviour [6–9], and are suggested to also vary in their metabolism [10,11]. In our study, breed was a powerful driver of variation in all studied measurands. However, the breed differences were highly specific across the different molecules, except in lipid measurands, including cholesterols, triglycerides, fatty acids and lipoproteins (figure 2). Heavy-structured and molossoid-type breeds BER, GR, LEO, AST and SBT generally had high levels of lipid measurands, and slender and muscular breeds GH, CHI, WH and PRT generally had low levels. Previously, higher lipid levels have been detected in some breeds when compared with the general canine population [14,17,18], and certain breeds are even predisposed to disorders of lipid metabolism, such as idiopathic hyperlipidaemia in Miniature Schnauzer, [31,56]. Although studies have been small and included limited number of breeds, body condition and the amount of body fat are demonstrated to differ between dog breeds [57–59]. Therefore, differences in the body composition and the amount of adipose tissue between the breeds could at least partially explain the observed systematic breed differences in lipid measurands. Additionally, heavy-structured breeds had high levels and slender and muscular breeds low levels of GlycA, except for CHI, who had a high GlycA concentration. GlycA is a composite inflammatory marker that consists of signals of different acute-phase proteins and is positively associated with systemic inflammation in humans [60–62]. Since glycosylated apolipoprotein slightly contributes to the GlycA signal, the concentration of circulating triglycerides can affect GlycA levels [61]. This may partly explain the high GlycA concentration in heavy-structured dogs. However, the triglyceride concentrations were low in CHI, suggesting other causes contributing to the high GlycA level observed in CHI.

Patterns in breed differences were not as clear in other metabolite subgroups. Additionally, even though the differences between the breeds with the highest and lowest measurand levels were relatively large across the different measurands, a breed-specific concentration exceeded the reference intervals only in creatinine. GH had remarkably higher creatinine concentration than other breeds, supporting a well-recognized and documented phenomenon [16,63–66]. Moreover, in the vast majority of the measurands, GH was systematically different from most other breeds. The other sighthound breed in our study, WH, imitated the measurand levels of GH. Sighthound breeds have been bred to race, which has resulted in physiological adaptations, such as larger muscle mass and higher amount of red blood cells [63], differentiating them from most of the breeds. Our results demonstrate that these physiological adaptations are reflected also in their metabolism and support the hypothesis that general reference intervals might not always be optimal for sighthound breeds.

Body size did not influence any measurand in our study as there was perfect multi-collinearity between breed and body size, and breed was a more important determinator of variation in the measurand levels. This probably results from the formation of the body size variable, as it was constructed based on the average heights of the breeds instead of actual measurements of the dogs, which were not available. However, as our results show quite large and systematic differences between the measurand levels of heavy-structured and lean breeds, it is possible that the actual body size and body condition could also influence metabolism. Metabolic differences between small and large-sized dogs have been described earlier [67,68], but breed effects have not been controlled in the studies. Notwithstanding, further research regarding the effects of actual height, weight and body condition of the study participants is warranted.

Age causes physiological and metabolic changes [58], and puppy and senior dogs are known to differ from adult dogs in certain clinical chemistry analytes [14,21–26]. Puppy (less than 1 year old), adult (1–7 years old), and senior (greater than 7 years old) dog-specific reference intervals have also been developed for the canine NMR platform [32]. Here, we investigated more thoroughly how the measurand levels change with age. Age significantly affected 112 of 119 measurands, and the majority of these effects were nonlinear. Additionally, most measurand levels increased with age, and 21 of 119 measurands, mostly lipids and GlycA, even exceeded the upper RL in dogs over 14 years old. In humans, GlycA is elevated in subclinical inflammatory conditions [61], which may be observed also in our study as subclinical conditions are relatively common in older dogs [25]. However, normal immunosenescent changes can also result in chronic low-grade inflammation, potentially increasing GlycA concentration [69]. Additionally, almost all cholesterols, triglycerides and lipoproteins showed the highest levels in old dogs. This may indicate age-related changes in lipid metabolism or reflect subclinical conditions [31] as several diseases, including cholestasis, chronic pancreatitis, infiltrative disease, hypothyroidism and inflammatory bowel disease are suggested to increase cholesterol levels in geriatric dogs [25].

Albumin and creatinine concentrations dropped below the lower RL in dogs older than 14 years, whereas the highest levels were observed in adult dogs. Our results are relatively consistent with previous research showing lower albumin [23,70,71] and creatinine [14,22,24,70,71] levels in puppies and young dogs than in adult dogs, and decreasing albumin [24–26] and creatinine [14,24] levels in old dogs. Creatinine concentration is affected by lean body mass [72,73] whereas low albumin level is predictive of poor muscle mass and may increase the risk of sarcopenia in the elderly [74]. Thus, the observed association between these measurands and age may reflect differences in the muscle mass of dogs of different ages. However, as albumin is also a negative inflammation marker [75], hypoalbuminaemia in old dogs may suggest subclinical conditions, similar to increased GlycA.

Our data suggest that female and male dogs have systematic metabolic differences, similar to humans [5,27,28], as 83 of 119 measurands differed between the sexes. Interestingly, the levels of most measurands, especially lipids, were higher in female dogs, which is supported by previous research showing higher cholesterol in females [23]. Sterilization and the interaction of sex and sterilization only affected a few measurands, including creatinine which was higher in neutered males than intact males whereas neutered and intact females did not differ from each other and had similar levels than intact males. Previously, elevated creatinine has been observed in male dogs and neutered dogs regardless of sex, as well as in human males [24,27]. This sex difference probably results from the greater proportional muscle mass of males [72]. One of the few measurands showing a higher level in male dogs was GlycA. Interestingly, opposite results have been obtained in humans [60]. Moreover, lower levels of the more traditional inflammation marker, C-reactive protein (CRP), have also been demonstrated in males [76], but in dogs, the current research suggests that CRP is not affected by sex [77].

Diet can have a profound influence on metabolism [29], evident also in our study. Even though the diet effects varied considerably, the highest measurand levels were usually observed in dogs eating solely dry food and the lowest levels in dogs eating solely raw food. Dogs eating solely raw food had the highest measurand concentrations only in a few measurands, including amino acids isoleucine, leucine, valine, total BCAA and BCAA/Tyr, the relative fatty acid measurands PalA%, SFA% and SteA%, and the Omega-6/Omega-3 fatty acid ratio. Dietary essential branched chain amino acid isoleucine, leucine and valine concentrations are highly dependent on diet in dogs [78]. These BCAAs are found in protein-rich foods, such as meat [79], similarly to the saturated fatty acids PalA and SteA. Thus, it is not surprising that dogs consuming a meat-based raw food diet had higher levels of these measurands. However, a discrepancy between the results of absolute and relative units of PalA, SteA and SFA must be acknowledged as dogs eating raw food had the highest levels of relative unit measurands PalA%, SteA% and SFA% but lowest levels of absolute unit measurands PalA, SteA and SFA. Previous studies have found lower serum cholesterol [80] and triglyceride [81] concentrations in dogs fed with raw food than in dogs fed with processed dry food diets, supporting our results showing systematically high levels of lipids in dogs eating dry food but low levels in dogs eating raw food. Nevertheless, the observed diet-related changes were relatively small. However, these results suggest that the NMR platform may be useful to identify, for example, aberrations in absorption and metabolism of nutrients, guiding attempts to find optimal diets for individual animals with specific needs.

As eating before blood sampling can cause unreliability and interference to the measurement of several analytes due to postprandial fluctuations and lipaemia [30,31], a 12 h fasting period before the sampling is a standard recommendation. Fasting time affected most measurands in the canine NMR platform, and the largest differences were commonly seen between dogs that had fasted less than 4 h and 12 h or more. Especially in glucose concentration, shorter fasting times resulted in significant glucose accumulation, indicating that an appropriate fasting protocol should be followed to maintain the integrity of the results [82]. However, in most other measurands, the observed differences between fasting times were generally relatively small.

The development, understanding and accurate interpretation of reference intervals requires information about physiological variation related to, for example, age and sex, since sometimes this variation can be so large that the population subgroups exceed or fall below the general reference intervals [83]. In this study, we examined the simultaneous effects of several physiological and diet-related variables on the canine serum measurand levels. With this approach, only age and breed had so large effects that the levels of certain measurands did not stay within the general reference intervals created for dogs of all ages. These results advocate the utilization of reference intervals determined for the age group in question. However, separate reference intervals cannot be created to account for all physiological factors, such as breed, or the interactions of several factors, as it requires infeasibly large sample sizes. Therefore, considering the effects of physiological variation currently relies on the knowledge of the clinician. Instead of traditional reference intervals, diagnostic algorithms that consider the possible simultaneous effects of multiple physiological factors, such as age, sex and breed of the individual, could provide personalized and more accurate ways to evaluate the clinical significance of the measured change for that particular individual.

Our study has limitations. First, the participants were not examined and verified as healthy by a veterinarian; instead, the health of the dogs was assessed with owner reports. Thus, subclinical conditions are possible, especially in aged animals. Additionally, we did not have information about the actual sizes (height at withers and weight) of the participants, which would have allowed more accurate investigation of the association between body size and measurand levels. Moreover, even though our initial sample size was quite high (4816 dogs) and included almost 200 different breeds, most breeds had too small sample sizes (less than 10) for statistical analyses, reducing the final sample size and number of studied breeds. In addition, the 95% confidence intervals were relatively wide for some measurands in certain explanatory variables, probably reflecting the small sample sizes of the corresponding variables, and indicates that the results must be interpreted cautiously. Finally, we had no information about the compositions of the dry food and raw food diets fed to the dogs, restricting the interpretation of the observed diet effects.

## 5. Conclusion

In this study, we investigated how breed, age, sex, sterilization, diet and fasting time influence the metabolite profiles of 2000 pet dogs. We showed that all these factors affect metabolism, but the effects were metabolite or metabolite group-specific. Especially age and breed caused outstanding variation in the measurand levels. In future, it is important to elaborate the causes why some measurands, including GlycA and several lipid measurands, increased dramatically in old dogs. Additionally, our results highlight the importance of controlling for physiological and diet-related factors in study designs and statistical analyses of metabolomics studies. In conclusion, this study provided notable information about the effects of normal physiology on canine metabolism, aiding accurate interpretation of laboratory results in veterinary diagnostics and serving valuable foundation for metabolomics studies of disease and physiological states.

## Data accessibility

The data presented in this study, together with relevant code for this research work, are stored in Dryad [84] (https://doi.org/10.5061/dryad.fj6q573w5). Additionally, the in-house model selection loop written in R is stored in GitHub: https://github.com/JNisk/airGLMs and has been archived within the Zenodo repository: https://doi.org/10.5281/zenodo.5839646 [38]. The data are provided in the electronic supplementary material [85].
